# Physiological triggers for beta-2 microglobulin amyloidosis: a view point from a biochemical prospective

**DOI:** 10.1093/ehjcr/ytad615

**Published:** 2023-12-09

**Authors:** Sukhdeep Kumar

**Affiliations:** Department of Medical Laboratory Sciences, School of Allied Medical Sciences, Lovely Professional University, Phagwara, Punjab 144411, India

I read with interest the article by Haslet *et al*.^1^ on a 63-year-old male with a rare cause of cardiac amyloidosis. This case report is an intriguing piece of research that has further implicated the role of mutations in beta-2 microglobulin (B2M) as the causal factor in cardiac amyloidosis. This report links the mutagenesis as a causal factor for a rare occurrence of B2M amyloidosis in the heart. The study confirmed the presence of B2M in amyloid deposit in cardiac tissue using mass spectrometry.

The study has only limitation that the possible effect of the Pro32Leu mutation was evaluated through bioinformatic analysis only, but there are many biophysical studies reviewed by Loureiro *et al*.,^2^ which also substantiate the role of mutations in the increased propensity of aggregation of B2M, as wild-type B2M has a negligible propensity to aggregate and attain amyloid conformations. This study has further consolidated the role of B2M in two different types of amyloidosis: one occurring during dialysis-related amyloidosis due to elevated levels of B2M and the other occurring due to mutations causing the B2M to become more aggregation prone and amyloidogenic.

While this study is consistent with the existing body of knowledge, we need a more integrative approach to understand the causal factors of B2M amyloidosis. A recent study has linked the B2M fragmentation and amyloidogenic changes under acidic conditions to a physiological response that mimics the stress conditions during microbial infection.^3^ In our research, we have found that B2M, even without any mutagenic changes, can undergo aggregation in the presence of a physiological concentration of calcium. These B2M microaggregates form larger aggregates and undergo amyloidogenic changes over 4 weeks of incubation.^4^

A possible mechanism of B2M deposition in the heart may be due to a combination of dysregulation of calcium homoeostasis, causing elevated levels of calcium in the cardiac tissue, which might be the trigger for genetic variants of B2M to aggregate and deposit in the heart. Another piece of research that supports this hypothesis is the study involving an 84-year-old male with myelomatosis, elevated calcium concentration, and impaired kidney function. The 18F-NaF positron emission tomography and computed tomography (PET/CT) scans indicated diffuse calcium deposition that might be linked to B2M aggregation and deposition in different tissues.^5^ Maintaining optimal calcium levels may improve the disease's prognosis and patient survival.

**Funding:** None declared.

**Ethics approval:** This article is based on previously conducted studies and does not contain any new studies with human participants or animals performed by any of the authors.

## Data Availability

All data are available from the corresponding author.
